# Complete mitochondrial genome of *Populicerus confusus* (Hemiptera: Cicadellidae: Idiocerinae)

**DOI:** 10.1080/23802359.2020.1788440

**Published:** 2020-07-23

**Authors:** Liang-Chen-Yu Shan, Hai-Rong Dong, Xiao-Chen Di, Li-Li Tian, Bin Zhang

**Affiliations:** a College of Life Sciences & Technology, Inner Mongolia Normal University, Hohhot, China; b Department of Clinical Laboratory, First Hospital of Hohhot, Hohhot, China

**Keywords:** Cicadellidae, Mitogenome, phylogeny; *Populicerus confuses*

## Abstract

In this study, we firstly reported the complete mitochondrial genome of *Populicerus confuses*. The complete mitochondrial genome was 16,395 bp in length which overall base composition was 41.43% A, 36.30% T, 11.54% C, and 10.73% G. It consisted of 13 protein-coding genes (PCGs), 22 tRNA genes, 2 rRNA genes (12S and 16S rRNA), and a control region (D-loop region). The complete mitochondrial genomes of *P. confuses* and other 9 species were used for phylogenetic analysis using the Bayesian method. The resulting phylogenetic tree confirms that the *Populicerus populi* is most closely related to *P. confuses*. The mitogenome provided the valuable evidence on phylogenetic relationship of the Idiocerinae at the molecular level.

Cicadellidae is a common component of all zoogeographic regions, with comprises 22,000 species in the world (Camisão et al. 2014). *Populicerus confuses* is a member of Idiocerinae subfamily. Idiocerinae is a moderately large, diverse group of arboreal leafhoppers, for which about 107 genera and 800 species are recorded worldwide (Zhang and Webb 2019). Currently, elucidating the structure of *P. confusus* mitogenome is important for understanding its diversity and evolution. The results will contribute for the identification and further phylogenetic analyses of the members of Idiocerinae.

In this study, we sequenced and analyzed the mitogenome of *P. confuses*, with the number of GenBank MT341642. The specimen was collected from Norjinkhairkhan, Khovd Province, Mongolia (N47.99, E91.62) on August 2019, and was deposited in the insect specimen room of Research Institute of Inner Mongolia Normal University (number IMNU2019081208). Genomic DNA isolated was sequenced using Illumina NovaSeq. The entire body without abdomen was shipped to Genepioneer (Nanjing, China) for genomic extraction. Sequencing was performed on an Illumina HiSeq 2000 instrument. The resultant reads were assembled using the SPAdes v3.10.1 (Bankevich et al. 2012). The complete mitochondrial genome was annotated with MITOS (Bernt et al. 2013). All tRNA genes were identified by tRNAscan-SE Search Server (Lowe and Chan 2016).

The complete mitochondrial genome of *P. confuses* was 16,395 bp in length with the A + T content of 77.73% (T 36.30%, C 11.54%, A 41.43%, and G 10.73%), which is within the range reported for hemipteran mitogenomes (68.86–86.33%; Zhang et al. 2014). It consisted of 13 PCGs (COX1-3, ND1-6, ND4L, ATP6, ATP8, and Cytb), 22 tRNA genes, 2 rRNA genes, and 1 D-loop region. Among the 13 PCGs, all genes took the start codon of ATN, while ATP8 and ND5 got TTG. The termination codon of these PCGs had two types (10 genes were TAA and 3 genes were single T––). In 13 PCGs, most genes are encoded on the H-strand, except for ND4, ND4L, ND5, and ND1. Eight tRNA genes (tRNA-Gln, Cys, Tyr, Phe, His, Pro, Leu1 and Val) are encoded on the L-strand. The 16S rRNA, with a length of 635 bp, is located between tRNA-Val and tRNA-Leu, and the 12S rRNA, with a length of 738 bp, is located between tRNA-Val and D-loop. The control region, with a length of 2107 bp, is located after 12S rRNA.

Phylogenetic analysis was carried out on the basis of 10 available mitogenomes of Cicadellidae insects in GenBank. It inferred from Bayesian inference using MrBayes v.3.2.1 (Ronquist and Huelsenbeck 2003; Ronquist et al. 2012) based on the PCGs of nine species of Idiocerinae and one outgroup. Sequences were aligned with MEGA6 software (Tamura et al. 2013). The phylogenetic tree showed that *Populicerus populi* is most closely related to *P. confuses* (Figure 1). The mitogenome provided the valuable evidence on phylogenetic relationship of the Idiocerinae at the molecular level.

**Figure 1.**
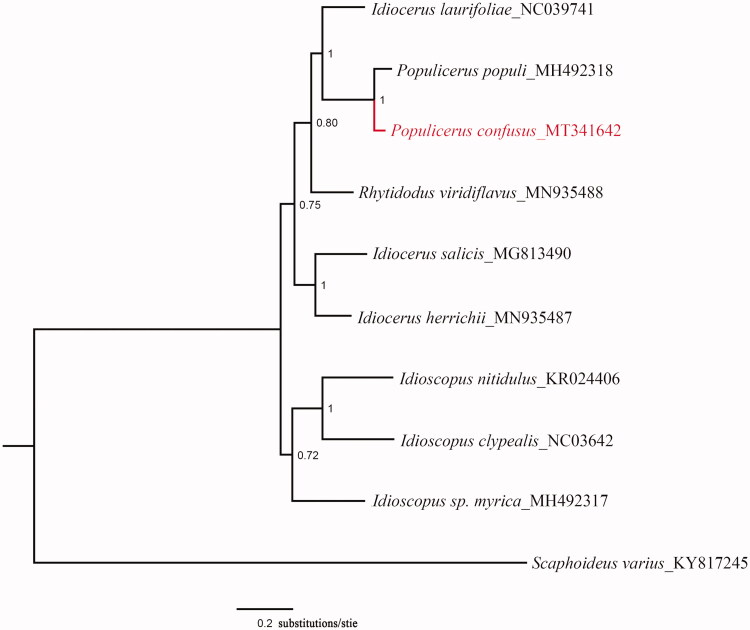
The phylogenetic tree which includes 9 Idiocerinae species and 1 outgroup using MrBayes v3.2.1 under the GTR + G model, based on the concatenated 13 PCGs. The posterior probabilities are labeled at each node. The GenBank numbers of all species are shown in the figure.

## Data Availability

The authors confirm that the data supporting the finding of this study are available within its supplementary material. https://www.ncbi.nlm.nih.gov/nuccore/MT341642
